# Effect of adding human chorionic gonadotropin to frozen thawed embryo transfer cycles with history of thin endometrium

**Published:** 2016-01

**Authors:** Robab Davar, Sepideh Miraj, Maryam Farid Mojtahedi

**Affiliations:** 1 *Department of Obstetrics and Gynecology, Research and Clinical Center for Infertility, Shahid Sadoughi University of Medical Sciences, Yazd, Iran.*; 2 *Shahrekord University of Medical Sciences, Shahrekord, Iran.*

**Keywords:** *Human chorionic gonadotropin*, *Embryo transfer*, *Endometrium*, *Fertilization*, *In-vitro*

## Abstract

**Background:**

Embryo implantation process is a complex phenomenon and depends on fetal and maternal factors interaction. Endometrial thickness is needed for successful implantation.

**Objective:**

We designed this study in order to assess adding human chorionic gonadotropin (HCG) to the conventional protocol in endometrial preparation in women with thin endometrium and a history of in vitro fertilization–embryo transfer (IVF-ET) failure.

**Materials and Methods:**

The non-randomized clinical trial study (quasi experimental design) was performed on 28 patients. Participants were women who were candidate for frozen-thawed (ET) and had two previous failed ET cycles because of thin endometrial. HCG was administrated (150 IU, intramuscular) from the 8th day of cycle and when endometrial thickness reached at least 7mm HCG was discontinued and frozen thawed ET was done.

**Results:**

Totally 28 patients were included. The mean ± SD age of participants was 30.39±4.7. The mean of endometrium thickness before and after HCG were 5.07±0.43 and 7.85±0.52, respectively p<0.001. Also, there were five clinically and chemically pregnant women.

**Conclusion:**

The findings of the study suggested that adding HCG to the conventional preparation method was an effective protocol and significantly improved endometrial thickness and pregnancy outcomes in women with previous embryo transfer failure because of thin endometrium.

## Introduction

Embryo implantation process is a complex phenomenon (1-3) and depends on fetal and maternal factors interaction. Endometrial thickness is needed for successful implantation (4-6). Sonographic endometrial parameters suggest that endometrial thickness less than 7 mm is associated with higher rates of failure. Therefore, endometrial thickness is a prognostic factor in implantation and a marker of endometrial receptivity (7-8). Increasing endometrial thickness makes a higher chance of clinical pregnancy (9-14). Patients who do not achieve at least 7 mm thickening are not good candidates for embryo transfer (ET). For improvement of endometrial thickness new therapeutic methods are suggested. For frozen-thowed ET, endometrium should be prepared by estrogen and progesterone (15). Patients with thin endometrium need additional estrogen. Current therapeutic methods for thin endometrium are: vitamin E, sildenafil, oral or vaginal estrogen, low dose of aspirin, pentoxifyllin and tocopherol (16-19). Adding other factors such as Granulocyte-colony stimulating factor (GCSF) infusion may improve endometrial thickness (20-22). Triple line method with thickening more than 7mm increase chance of successful implantation greater. 

Secretion of human chorionic gonadotropin (HCG) is one of the first embryonic signals. Also, HCG is the embryo-endometrial relationship regulator (23). HCG controls the implantation and embryonic development (24). We designed this study in order to assess adding HCG to the conventional protocol for endometrial preparation in women with thin endometrium and history of implantation failure.

## Materials and methods

This non-randomized clinical trial was approved by the Ethics Committee of Shaheed Sadoughi University of Medical Sciences. This study was performed from September 2013 to January 2014. Participants were selected among women who were candidate for frozen-thawed ET. All of patients had previous failed IVF cycle due to thin endometrium. The written consent was obtained from all of the participants. The inclusion criteria were: patients with at least 2 previous failed IVF cycle, thin endometrium (thickness <7 mm) despite conventional treatment in previous IVF cycles, pregnancy failure despite vaginal estrogen, sildenafil, vitamin E. Patients with intrauterine lesion such as sub mucosal leiomyoma, history of endometriosis, and thyroid disorders were excluded. (In this study the endometrial thickness was assessed before and after HCG treatment, the endometrial hickness in the last IVF cycle was documented as before experimental findings).

All the patients received 8 mg estradiol valerate from the second day of menstrual cycle and continued at least for seven days. HCG (Pregnyl®, Darou Pakhsh, Iran) was administrated (150 IU, intramuscular) from the 8^th^ day of cycle. The HCG vial (1500 IU) was diluted 10 times and one was injected every day. On the 12^th^-13^th^ day trans-vaginal sonography was done. Endometrial thickness was measured by a blinded gynecologist. If endometrial thickness was proper, (at least 7mm) HCG stopped and after 24 hr progesterone 100 mg intramuscular was injected for 3 days. Finally, ET was performed. The mean number of ET was 2.12 ± 0.56. The pregnancy test (serum βHCG) was done two weeks after ET. Fetal heartbeat (clinical pregnancy) was checked after two weeks.

Clinical outcomes included: increase of endometrial thickness more than 7 mm, improvement of endometrial thickness >10% and >20%, βHCG titer more than 25 mIU/ml was considered as chemical pregnancy. Heart beats existence two weeks after chemical pregnancy was considered as clinical pregnancy.

**Statistical analysis**

All statistical analysis was done by the SPSS software (Statistical Package for the Social Sciences, version 20; SPSS Inc, Chicago, Illinois). The study sample size was calculated according to the “comparison the means” formula. The accepted differences before and after the trial was 0.5mm (α=0.05) and the study power was 0.9. The normal distribution of data was checked. Mean, standard deviations, minimum and maximum were calculated. Paired t-test was used to compare the endometrial thickness before and after trial. The statistical significances considered as 0.05.

## Results

**Figure 1:**
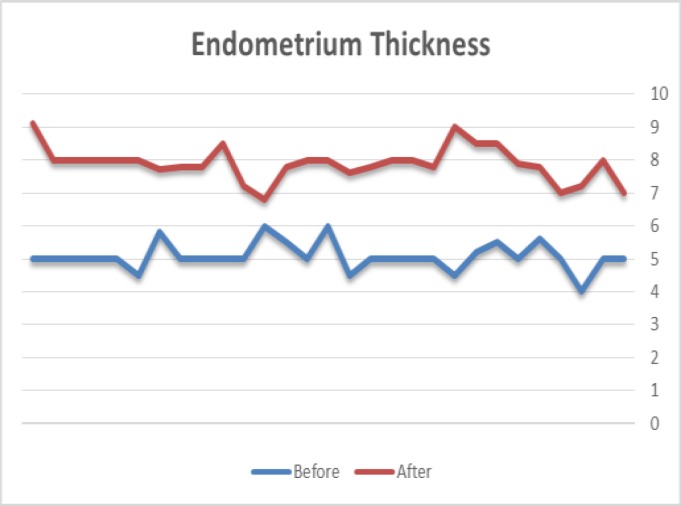
Endometrial thickness before and after HCG treatment

Totally, 28 patients were included. The mean age of the participants was 30.39 ± 4.70 years old. The youngest patient was 22 and the oldest was 41 years old. None of the patients had the history of chemical or clinical pregnancy. Before and after adding HCG, the mean scores of endometrial thickness in the frozen-thawed ET cycles were 5.07±0.43mm and 7.85±0.52 mm, respectively, which was significantly increased (Table I, figure 1). Although there were 5 clinical and 5 chemical pregnant women, but the mean score of endometrial thickness was not different in pregnant and non-pregnant women (p-value= 0.856). 

**Table I:** Participant’s outcome after HCG treatment

**Variable**	**Before HCG**	**After HCG**	**p-value**
10% improvement n (%) 1	-	26(92.9)	Non applicable 2
20% improvement n (%)	-	25(89.3)	Non applicable
Endometrial thickness* (mean±SD)	5.07±0.43	7.85±0.52	< 0.001
Clinical pregnancy n (%)	No	5(17.8)	Non applicable
Chemical pregnancy n (%)	No	5(17.8)	Non applicable

HCG: Human chorionic gonadotropin

*: Paired t-test was used

1 : The improvement percentage was calculated by dividing endometrial thickness

2 : Since there was no improvement or clinical pregnancy before adding HCG the comparison is not applicable.

## Discussion

Thin endometrium is one of the major causes of assisted reproductive therapy failure (1-6). Proper endometrial thickness is necessary for successful embryo implantation and increasing rate of pregnancy (7-14). The insufficient thin endometrium is an unresolved clinical problem (21). Several treatment protocols are suggested for endometrial responsiveness in women who are candidates for IVF including aspirin (low dose), sildenafil citrate, pentoxifyllin, vitamin E (16-19, 22). The traditional treatment for increasing endometrial thickness was the increase dose of estrogen supplement. Sildenafil can improve endometrial blood supply and thickness in patients with a history of poor endometrial responsiveness (25-26). Combination of pentoxifyllin and vitamin E is an effective method for endometrial thickness and it can increase pregnancy rates (27).

Several biologic studies suggest that GCSF can be considered as an effective treatment protocol, which can improve IVF outcomes in women with thin endometrium and a history of repeated embryo implantation failure (2, 20, 21, 26). Recent studies tested adding HCG plus conventional endometrial preparation protocol (27-31). Eftekhar et al. showed that HCG supplementation for endometrial preparation benefit is equal with estradiol and progesterone (23). The current study revealed that adding HCG to the conventional endometrial preparation protocol was effective in women with an IVF failure history and thin endometrium (30). Tesarik et al. suggested HCG supplementation during the luteal phase of oocyte donation cycle might improve the pregnancy rate (31). Adding HCG improved endometrial thickness, chemical and clinical pregnancy in women with an IVF failure history and thin endometrium. HCG supplementation in luteal phase could improve pregnancy rate (32). Intrauterine injection of HCG before ET increased successful rate of pregnancy in ART (33). HCG plays a critical role in the initiation and maintenance of pregnancy. Adding HCG during the secretory phase modulated several endometrial paracrine parameters, which correlate to endometrial differentiation (IGFBP-1), angiogenesis (VEGF), implantation (LIF, M-CSF) and tissue remodeling (MMP-9) (34). The before- after method which was used in this study, can be considered as a strength point of study. The before-after design eliminated the confounders. Although the findings showed significant differences between before and after adding HCG cycles, but the small sample size may be the study limitation. For future considering, a systemic review and meta-analysis may clear the way.

The findings of this study suggested that adding HCG to the conventional preparation method was an effective protocol and significantly improved endometrial thickness and pregnancy outcomes in women with previous implantation failure due to thin endometrium.

## Note

***This article extracted from fellowship course thesis. (*** ***Sepideh Miraj)***

***Registration ID in IRCT: IRCT201512082604N2***
